# Loss of γ-aminobutyric acid D-Type Motor Neurons in Young Adult *Caenorhabditis elegans* Following Exposition with Silica Nanoparticles

**DOI:** 10.3390/cells14030190

**Published:** 2025-01-27

**Authors:** Dang Tri Le, Stella Pauls, Gereon Poschmann, Kai Stühler, Anna von Mikecz

**Affiliations:** 1 Leibniz Research Institute of Environmental Medicine GmbH (IUF), Auf’m Hennekamp 50, 40225 Düsseldorf, Germany; 2 Institute of Molecular Medicine, Proteome Research, Medical Faculty and University Hospital, Heinrich Heine University Düsseldorf, 40225 Düsseldorf, Germany; 3 Molecular Proteomics Laboratory, Biomedizinisches Forschungszentrum (BMFZ), Heinrich Heine University Düsseldorf, 40225 Düsseldorf, Germany

**Keywords:** air pollution, *C. elegans*, GABA, GABAergic neurons, silica nanoparticles, tire wear

## Abstract

Although *Caenorhabditis elegans* is commonly used to assess the neurotoxicity of environmental pollutants, studies that explore the intricate biology of its nervous system, particularly those addressing long-term effects and aging in adult worms, are rare. These models offer significant advantages for understanding the full spectrum of neurobiological impacts. Here, we investigated the effects of silica nanomaterials on the γ-aminobutyric acid (GABA) neural system in young to middle-aged nematodes and found a unique degeneration pattern characterized by loss of anterior- and posteriormost GABAergic D-type motor neurons. Four-day-old nematodes were identified as a vulnerable age group, where the pollutant-accelerated neurodegeneration that is typically seen in old *C. elegans*. Proteomics of 4-day-old *C. elegans* revealed significant alterations of protein abundance, including the downregulation of proteins such as glutamate dehydrogenase (gdh-1) and glutamate oxaloacetate transaminase (got-1.2), which are essentially involved in GABA metabolic pathways. Consistent with these findings, we demonstrated locomotion deficits in *C. elegans* exposed to nanoscale silica by establishing a semi-automated behavioral arena. Our setup not only visualizes but also automatically quantifies vulnerabilities at the individual worm level. This novel neurodegeneration model now enables the simulation of real-world pollutant mixtures and environmental conditions, capturing the complexity of the exposome.

## 1. Introduction

Recent projections indicate that there are around 350,000 chemicals currently being produced and used worldwide [1]). Nanomaterials such as silica nanoparticles (NPs) have been industrially produced since the middle of the 20th century and are extensively used in a wide range of products, including varnishes, pesticide additives, and car tires [2]. When car tires wear down over time, rubber and its additives, including silica NPs, are released into the environment as tire wear. Silica NPs contribute to air pollution as part of particulate matter (PM) and accumulate in environmental sinks like air, water, sediments, and soil. Modeling approaches estimate the environmental dispersion of silica NPs in the European Union, revealing median concentrations of 0.12 μg/L of nano-silica in surface waters and an annual increase in sediments of 0.43 mg/kg [2].

To keep pace with rapid advancements in nanotechnology and the dispersal of nanomaterials into the environment, innovative methodologies are needed to elucidate bio-interactions. These methods should address both the biological responses to NPs and the safe application of nanotechnology. *C. elegans* is valuable for this task because, as a bacterivore in its natural soil habitat, it is a realistic target for soil pollutants, including silica NPs [3]. In the laboratory, it is well-suited for the study of nanomaterial interactions with the nervous system, in particular the exploration of molecular pathways of neurodegeneration and the assessment of neurobehavioral responses. Its short lifespan, whole-life analysis capability, and ease of maintenance make *C. elegans* ideal for investigating nano–bio interactions of neurotoxic nanomaterials, particularly in relation to adult age [4].

The nervous system of *C. elegans* is a well-defined network comprising 302 neurons in the adult hermaphrodite, organized into a central nerve ring (the brain) and longitudinal nerve cords [5]. This system’s simplicity, along with its fully mapped neural circuitry and nearly complete expression maps for neuronal receptors and other molecules, facilitates detailed research [6,7]. Sensory neurons detect external stimuli, interneurons process this information, and motor neurons regulate muscle contractions and movement. Chemical screens in *C. elegans* enable researchers to correlate gene function with neurotransmission and circuit-level activity.

Neurotransmitters in *C. elegans* include dopamine, serotonin, and γ-aminobutyric acid (GABA). In nematodes, GABA acts at neuromuscular synapses, regulating motor functions such as muscle relaxation during locomotion and foraging [8,9]. The D-type motor neurons, consisting of 6 dorsal D-type (DD) and 13 ventral D-type (VD) neurons, play a key role in this process. Located in the ventral nerve cord, they innervate the dorsal and ventral body muscles, respectively, and inhibit muscle contraction on one side of the body while the opposite side contracts [10]. This GABA-mediated inhibition allows for the characteristic sinusoidal movement of the worm on solid ground by enabling contralateral muscle relaxation during body bending. In the absence of DD and VD neurons, worms lose the ability to relax their body wall muscles, resulting in unopposed excitatory input and reduced wave amplitude during locomotion [8,9]. However, they can still adopt a sinusoidal posture and can swim though with diminished movement efficiency. Models of neurodegeneration in GABAergic neurons, using genetic or environmental manipulations to induce selective degeneration, enabled researchers to study neurodegenerative mechanisms and potential therapies. The neuroprotective effects of compounds with GABAergic activity, such as propofol, thymol, and chlorothymol, have been demonstrated, as they help protect neurons from damage by enhancing GABA-mediated inhibitory signaling and reducing excitotoxicity [11,12].

In this study, we investigated the effects of silica nanomaterials on the GABAergic neural system of *C. elegans* reporter nematodes, which fluorescently label all GABA neurons. Nano-silica specifically induced a loss of anterior- and posteriormost D-type GABA motor neurons, which correlated with neurobehavioral defects of forward locomotion. To visualize individual behavior and perform automated quantification, we established a single worm-tracking platform. This platform enabled the identification of Aerosil 90 and 200, components of tire wear and other consumer products, as neurotoxicants in *C. elegans*. On the molecular level, proteins involved in the metabolic pathway of GABA synthesis were under-expressed in nematodes exposed to silica NPs, potentially contributing to neurodegeneration, the loss of neurons, and related behavioral defects.

## 2. Materials and Methods

### 2.1. Particles

Precipitated silica NPs with diameters of 50 nanometers (nm) and BULK silica particles with diameters of 500–1000 nm were obtained from Kisker (Steinfurt, Germany) and Sigma-Aldrich (Darmstadt, Germany), respectively. Fumed silica NPs with diameters of 12 nm (Aerosil 200) and 20 nm (Aerosil 90) were obtained from Evonik Industries (Essen, Germany). Stock solutions of silica particles were prepared by suspending 25 mg/mL powder in distilled water (H_2_O). The properties of silica particles were analyzed using live cell imaging, transmission electron microscopy (TEM), and dynamic light scattering, as described and listed in previous studies [13].

### 2.2. C. elegans Strains

*C. elegans* strains were maintained at 20 °C on nematode growth medium (NGM) plates supplemented with yeast extract, and fed with live *E. coli* strain OP50. The strains used included wild-type Bristol N2, serotonergic neuron reporter strain LX975 vsls97 [tph-1p::DsRed2 + lin-15(+)], dopaminergic neuron reporter strain BZ555 egls1 [dat-1p::GFP], GABAergic neuron reporter strain EG1285 oxIs12 [unc-47p::GFP + lin-15(+)], and muscle cell reporter RW1596 myo-3(st386) V; stEx30 [Pmyo-3::GFP+ rol-6(su1006)]. All strains were acquired from the *Caenorhabditis* Genetics Center (University of Minnesota, Minneapolis, MN, USA).

### 2.3. Particle Exposures in Liquid Media

Nematodes were synchronized by isolating eggs using hypochlorite/NaOH treatment and allowed to hatch on NGM plates at 20 °C. At the L4 larval stage, nematodes were transferred to 96-well plates containing liquid medium (S-medium, pH 5.7, and 0.1 µg/mL fungizone) with 12 mg/mL freshly prepared *E. coli* OP50 [14]. For age-resolved experiments in young and middle-aged *C. elegans*, nematodes were treated with 5-fluoro-2′-deoxyuridine (FUdR, 1.5 mM final concentration) to maintain synchronization. On day 1 of adulthood, worms were either mock-treated with H_2_O or exposed to silica NPs or BULK silica at the indicated concentrations. All experiments were performed at 20 °C.

### 2.4. Microscopy and Quantification of Neurodegeneration

Living reporter nematodes were analyzed from young (day 2) to middle-aged (day 11) on 5% agarose pads with 10 µM NaN3 at room temperature. Micrographs were captured with a stereo microscope (SMZ18, Nikon Instruments Europe, Amsterdam, The Netherlands) with an SHR Plan Apo 2x objective. For fluorescence detection, the reporter DsRed2 was visualized with 561 nm excitation/575–615 nm emission. The reporter green fluorescent protein (GFP) was detected with 488 nm excitation/510–550 nm emission. Image processing was conducted using the NIS Elements software version 5.21.03 (NIKON Instruments Europe, Amsterdam, The Netherlands). Neurodegeneration in single neurons was categorized as complete loss of fluorescent signal (staining of somata) in GABAergic neurons. Dopaminergic or serotonergic neurons were examined for a discontinuous dotted staining pattern along dendritic processes and axons, which was quantified as beading.

### 2.5. Behavior Assays

Manual thrashing assay: *C. elegans* were either mock-treated or exposed to 200 μg/mL silica NPs in liquid culture. Nematodes aged 2 to 11 days were transferred from 96-well microplates to 24-well microplates. Swimming behavior in the liquid medium was quantified by manually counting the thrashes of individual worms over a period of 30 s. Swimming videos were captured using a Nikon SMZ800 microscope equipped with a DS-Fi2 digital camera and recorded with NIS Elements software version 5.21.03 (Nikon Instruments Europe, Amsterdam, The Netherlands).

Single-worm tracking (SWT): *C. elegans* were either mock-treated or exposed to 200 μg/mL silica NPs in liquid culture within 96-well plates. Nematodes aged 2 to 11 days were transferred from 96-well microplates to 6-well agar plates overlaid with 2 mL of M9 buffer. Swimming phenotypes were recorded using a Nikon SMZ800 microscope equipped with a DS-Fi2 digital camera, and the videos were captured with NIS Elements software version 5.21.03 (Nikon). The thrashing tracks of individual worms were visualized, and the thrashes were quantified by the automated SWT system [15].

### 2.6. Sample Preparation for Proteomic Analysis

GABAergic reporter *C. elegans* (strain EG1285) was transferred as larval stage L4 to 6-well plates containing liquid medium, 20 mg/mL *E. coli* OP50, and FUdR (1.5 mM final concentration) to maintain age-synchronization. On day 1 of adulthood, nematodes were either mock-exposed (H_2_O) or exposed to 200 µg/mL silica NPs. On day four of adulthood, the worms in each group were collected in cryotubes, washed with M9, spun down, and pellets stored in liquid nitrogen. Six independent experiments were conducted with 8000–12,000 worms per condition per experiment.

### 2.7. Worm Lysis, Protein Determination, and PAA Gel Quality Control

Protein lysates were prepared by bead milling of pelleted worms in a lysis buffer containing urea, thiourea, and CHAPS (3-[(3-Cholamidopropyl)dimethylammonio]-1-propanesulfonate), as previously described [16]. The protein concentration of the resulting samples was then measured using the Pierce 660 nm protein assay (Thermo Fisher Scientific, Waltham, MA, USA). For quality control, proteins were separated using 4–12% Bis(2-hydroxyethyl)amino-tris(hydroxymethyl)methane polyacrylamide gels (Novex NuPAGE, Thermo Fisher Scientific) and visualized by silver staining.

### 2.8. Protein Sample Preparation for Mass Spectrometric Analysis

Sample preparation for mass spectrometric analysis was carried out by a modified version of the single-pot, solid-phase-enhanced sample preparation (SP3) protocol [17]. Briefly, 10 µg protein was prepared in 35 µL lysis buffer, then 5 µL 10% (*w*/*v*) sodium dodecyl sulfate in water was added followed by 5 µL 100 mM dithiothreitol. Proteins were reduced for 20 min at 56 °C and alkylated by adding 6.7 µL 300 mM iodoacetamide and incubation for 15 min at room temperature. A total of 5 µL of a 20 µg/µL bead mixture (Sera-Mag SpeedBeads GE45152105050250 and GE65152105050250, Merck, Darmstadt, Germany) was added, and proteins were precipitated by adding 160 µL ethanol for 15 min. Beads were washed three times with 200 µL 80% ethanol and 200 µL acetonitrile. For overnight digestion, proteins were resuspended in 18 µL 50 mM triethylammonium bicarbonate buffer including 0.31 µg trypsin (Serva, Heidelberg, Germany), and incubated at 37 °C while shaking. On the next day, another 0.31 µg trypsin was added and the sample was incubated for another 4 h. Five hundred nanograms of protein per sample were prepared in a total volume of 17 µL 0.1% trifluoroacetic acid for mass spectrometric analysis.

### 2.9. Mass Spectrometric Analysis of the C. elegans Proteome

Prepared peptides were separated over 2 h using an UltiMate 3000 Rapid Separation Liquid Chromatography (RSLC) system (Thermo Fisher Scientific) on C18 material, as previously described [16]. The separated peptides were then ionized via a nano-source interface and analyzed by a Fusion Lumos mass spectrometer (Thermo Fisher Scientific) equipped with a FAIMS device (compensation voltage was set to −50 V) in data-independent positive mode. First, survey spectra were recorded in the orbitrap (resolution: 60,000, scan range 380–985 *m*/*z*, maximum injection time 100 ms, automatic gain control target: 400,000, profile mode), and second, precursors were isolated in 2 × 30 slightly overlapping 10 *m*/*z* windows in the mass range from 385 to 981 *m*/*z*, fragmented by higher-energy collisional dissociation (collision energy 30%), and analyzed in the orbitrap (resolution: 15,000, scan range 145–1450 *m*/*z*, maximum injection time 40 ms, automatic gain control target: 100,000, centroid mode). The analysis of mass spectrometric data was carried out with DiaNN 1.8.1 [18] which was used for peptide and protein identification and quantification with standard parameters, if not stated otherwise. *C. elegans* protein sequences from the UniProt knowledge base (UP000001940, downloaded on 12 January 2023, 26,728 sequence entries) as well as potential contaminants (sequences from MaxQuant 2.1.0.0) were used for the searches. Methionine oxidation was additionally considered as a variable modification. Only identified proteins showing a q-value on PSM and protein group level of <0.01 were included. The mass spectrometry proteomics data have been deposited to the ProteomeXchange Consortium via the PRIDE (PMID 34723319) partner repository with the dataset identifier PXD056717. Quantitative protein data was analyzed using the Perseus framework (version 1.6.6.0, Max Planck Institute for Biochemistry, Planegg, Germany). Proteins were included in the analysis only if they had at least 2 valid quantitative (LFQ) values in both investigated groups. Prior to statistical analysis, normalized (LFQ) intensities were log2-transformed Differentially abundant proteins were identified using the Student’s *t*-test-based significance analysis of microarrays (SAM) method [19], with an S_0_ parameter of 0.1 and a false discovery rate (FDR) of 5%.

### 2.10. Statistical Analyses

Data are presented as mean ± SD from at least three independent biological replicates unless stated otherwise. Violin plots, bar graphs, and line graphs were created in OriginPro 2022 (OriginLab Corporation, Northampton, MA, USA). The weighted dot plots were generated using R programming (R 4.4.2, R-project.org). For thrashing rates, normality was assessed by the Shapiro–Wilk test. Depending on the normality of the data distribution, statistical differences were determined using the Kruskal–Wallis test with Dunn’s post hoc test or one-way ANOVA with Tukey’s post hoc test. Statistical analyses were performed in OriginPro 2022 (Origin Lab Corporation). *p* values of <0.05 were considered statistically significant (*, *p* < 0.05; **, *p* < 0.01; ***, *p* < 0.001, ****, *p* < 0.0001).

## 3. Results and Discussion

### 3.1. Silica NPs Induced Loss of GABAergic D-Type Motor Neurons

**Figure 1:**
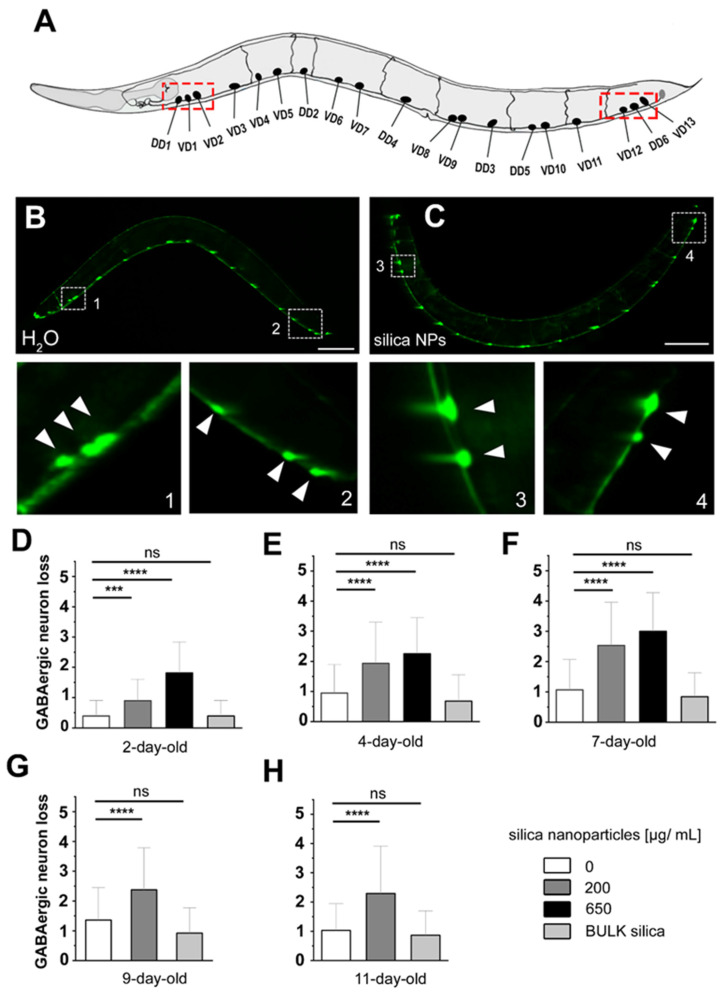
Silica NPs induce loss of soma fluorescence in GABAergic D-type motor neurons. (**A**) Scheme of the positions of the GABAergic D-type motor neurons in the adult hermaphrodite nematode. Red boxes indicate the anteriormost and posteriormost GABAergic neurons, which are susceptible to exposure to silica NPs. (**B**,**C**) Representative fluorescent micrographs of 4-day-old adult *C. elegans* reporters stably expressing green fluorescent protein (GFP), under the control of the *unc-47* promoter in all GABAergic neurons (strain EG1285). (**B**) Reporter worms were mock-treated with H_2_O, or (**C**) exposed to 200 µg/mL silica NPs. Insets show blow-ups of anteriormost and posteriormost GABAergic D-type motor neurons. Arrows indicate the respective cell bodies (somata). (**D**–**H**) Quantification of GABAergic neuron loss in 2- to 11-day-old worms that were either mock-exposed or exposed to 200 µg/mL silica NPs, 650 µg/mL silica NPs, or 200 µg/mL BULK silica. Bar graphs represent means ± SD from three independent experiments, with *n* = 25 per condition per experiment (one-way ANOVA with Tukey’s post hoc test). Statistical significance is denoted as follows: ***, *p* < 0.001; ****, *p* < 0.0001. BULK, silica particles with a diameter larger than 100 nanometers; H_2_O, distilled water; ns, not significant; s, seconds; SD, standard deviation.

In the nematode *C. elegans*, GABAergic motor neurons play an important role in forward locomotion. To examine the impact of silica NPs on the GABAergic nervous system, reporter worms expressing GFP under the control of the *unc-47* promoter in GABAergic neurons were investigated (Figure 1). These reporter worms were either mock-exposed (H_2_O) or exposed to silica NPs or BULK silica for analysis. In control worms all GABAergic neurons were visualized, showing a bead-like fluorescent pattern along the ventral side of the worm, with each bead representing the soma of a GABA neuron (Figure 1B). The insets show the three anteriormost and three posteriormost D-type motor neurons (arrows). After exposure to 200 µg/mL silica NPs, a fluorescence loss in respective D-type motor neuron soma was observed in the anteriormost and posteriormost regions (Figure 1C, insets).

Next, the loss of soma fluorescence was quantified in young to middle-aged GABA neuron reporters (Figure 1D–H). In 2-day-old worms, exposure to 200 µg/mL silica NPs significantly decreased the number of fluorescent GABA neuron soma compared to controls (*p* < 0.001), with an even greater effect at a higher concentration of 650 µg/mL (Figure 1D). The mean fluorescent neural soma loss increased from 0.5 in controls to approximately 1 in the 200 µg/mL silica NP-exposed group and nearly 2 in the 650 µg/mL group. In 4-day-old worms, mean fluorescent neural soma loss reached about 2.5 and 3 in the 200 µg/mL and 650 µg/mL groups, respectively (*p* < 0.0001), peaking in 7-day-old worms. For older worms (9-day-old and 11-day-old), neuronal soma loss remained around 2.5 in the 200 µg/mL group. However, the 650 µg/mL silica NP concentration proved lethal and could thus not be determined. In contrast, BULK silica did not cause significant neurodegeneration at any age, suggesting that the observed toxicity is due to the nanoscale properties of silica particles. We concluded that silica NPs induce a novel kind of neurodegeneration which is characterized by loss of anteriormost and posteriormost GABAergic D-type motor neurons. Generally, the loss of the complete soma is considered the final stage of neurodegeneration that is preceded by dotted fluorescence of dendrites [20,21,22]. Notably, such dotted dendritic patterns were observed not in GABA neurons but in dopaminergic and serotonergic neurons following exposure to silica NPs ([13]; Figures S1–S4). This is consistent with the idea that while nanoscale silica particles induce pan-neuronal neurodegeneration, anteriormost and posteriormost GABAergic D-type motor neurons are specifically vulnerable and subjected to complete loss.

To determine if the observed loss of soma fluorescence in GABA neurons reflects actual degeneration and cell death, we analyzed the abundance of proteins involved in apoptosis by mass spectrometry-based proteomics in unexposed and nano-silica-exposed 4-day-old reporter worms expressing GFP under the control of the *unc-47* promoter in GABAergic neurons. Specifically, the genes *ced-6*, *lst-4*, and *pig-1*, which are critically involved in the engulfment and degradation of apoptotic cells, exhibited higher abundance (Figure S5). We conclude that neurotoxicity induced by silica NP exposure in *C. elegans* involves neuronal loss attributable to cell death mechanisms, including apoptosis.

Given the crucial role of GABA neurotransmission in *C. elegans* forward locomotion and neural circuit stability, a reduction in GABAergic neurons could disrupt the balance of muscle excitation and inhibition. The balance between excitatory acetylcholine and inhibitory GABA signals ensures the proper coordination of muscle contractions necessary for movement [10]. Loss of GABAergic D-type motor neurons and respective inhibitory neurotransmission may disturb this balance and result in impaired crawling and swimming locomotion.

### 3.2. Silica NPs Reduce C. elegans Locomotion

**Figure 2:**
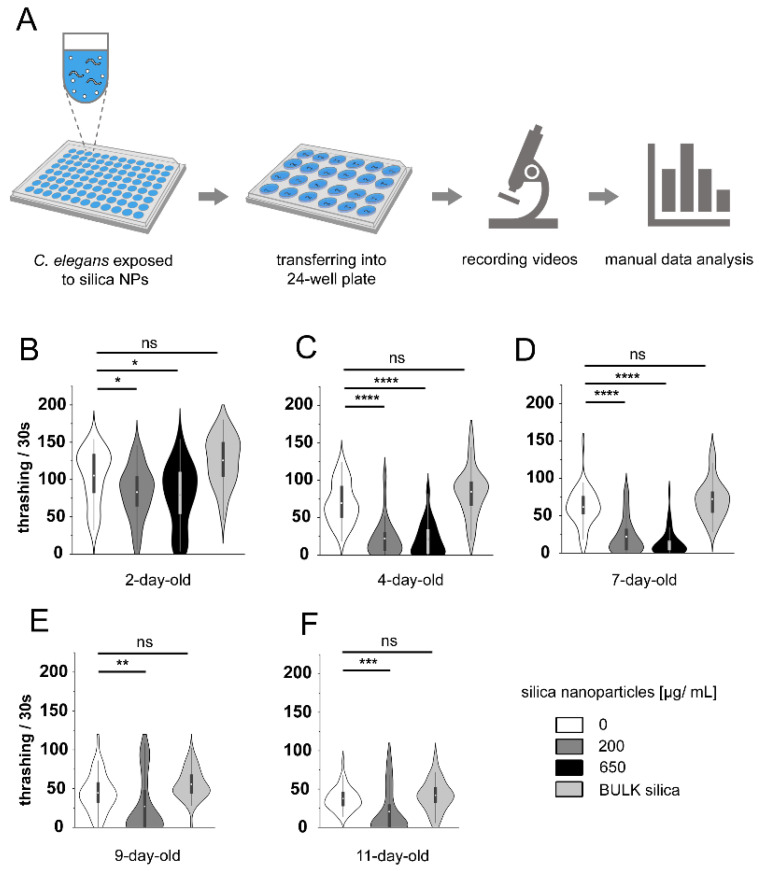
Silica NPs modulate neurobehavior in young and middle-aged GABAergic reporter worms. (**A**) The schematic outlines the sequential steps of a protocol involving the exposure of *C. elegans* to pollutants in liquid media, video microscopy to observe locomotion phenotypes, and manual quantification. Adult hermaphrodite *C. elegans* reporters for GABAergic neurons (strain EG1285) were mock-exposed to H_2_O or exposed to increasing concentrations of silica NPs or larger silica particles (BULK) in 96-well microtiter plates. Young, middle-aged, and old worms were placed in 24-well plates for video recording. The videos were analyzed manually. (**B**–**F**) Violin plots represent quantification of the locomotion phenotype swimming in 2- to 11-day-old worms. Swimming was quantified as the thrashing rate per 30 s. Worms were either mock-treated or exposed to silica NPs (200 μg/mL, and 650 μg/mL), or BULK silica (200 μg/mL). Violin plots display means ± SD from three independent experiments, with n = 15 worms per condition per experiment. Statistical significance is indicated as follows: *, *p* < 0.05; **, *p* < 0.01; ***, *p* < 0.001; ****, *p* < 0.0001. BULK, silica particles with a diameter larger than 100 nanometers; NPs, nanoparticles; ns, not significant; s, seconds; SD, standard deviation.

To test the hypothesis that silica NP-induced loss of GABAergic D-type motor neurons plays a role in locomotion, we tested the swimming phenotype in young and middle-aged reporter worms for the *C. elegans* GABA nervous system (Figure 2). The thrashing rate over a time period of 30 s was analyzed in young to middle-aged worms using a behavior arena on 24-well plates. Following 24 h of exposure, the control group demonstrated a high mean thrashing rate of 105 thrashes per 30 s, indicative of robust neuromuscular function. In contrast, exposure to 200 µg/mL silica NPs significantly reduced this rate to 82 thrashes (*p* < 0.05) (Figure 2B), with a further decline to 79 thrashes at 650 µg/mL (Figure 2C). Importantly, exposure to BULK silica did not affect the thrashing rates at any age, as these remained comparable to those of the mock control group. From day 4 onwards (Figure 2B–F), all groups exhibited a natural decline in thrashing rates with age. The most pronounced reduction was observed in 7-day-old worms exposed to 650 µg/mL silica NPs, which had an average thrashing rate of only 14 thrashes per 30 s, compared to 21 thrashes in the 200 µg/mL group and 61 thrashes in the control group (*p* < 0.0001). Although untreated 11-day-old worms exhibited a lower thrashing rate (37 thrashes) compared to 2-day-old worms (105 thrashes), they still maintained a higher rate than the 200 µg/mL silica NP-treated worms at the same age, which showed a mean rate of 20 thrashes per 30 s (*p* < 0.001). These results underscore significant, dose-dependent, and age-dependent neuromuscular deficits induced by silica NPs, directly correlating with the observed loss of GABAergic neurons. The substantial reduction in thrashing rates, particularly at higher NP concentrations and in older worms, aligns with the neurodegeneration of GABAergic neurons. This neuronal loss may critically impair the inhibitory functions of GABA, leading to disrupted neuromuscular activity and diminished motor control.

### 3.3. Visualization and Automatic Quantification of Silica NP-Induced Locomotion Deficits

**Figure 3:**
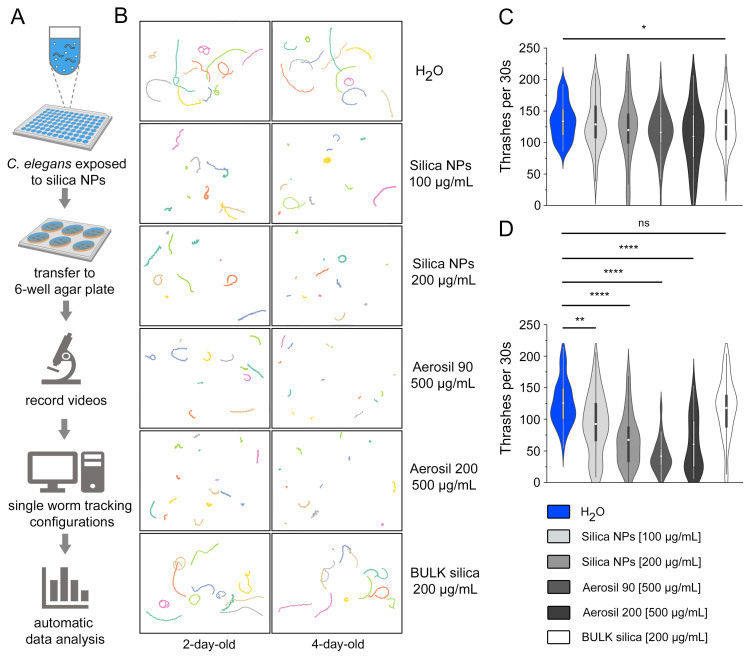
Single-worm tracking visualizes reduction in locomotion by silica NPs. Software for single-worm tracking (SWT) was applied to various particle types, concentrations, and worm ages. (**A**) The schematic outlines the sequential steps of a protocol involving automated data analysis in *C. elegans* by means of SWT. Adult hermaphrodite *C. elegans* (N2) are left unexposed or exposed to silica particles in liquid culture within 96-well plates. Two-day-old or four-day-old nematodes from individual wells of a 96-well plate were transferred and dispersed into separate wells of a 6-well plate containing solid agar for video recording. (**B**) Representative thrashing maps of individual worms generated by the automated SWT system (multicolored tracks). Young *C. elegans* were either mock-exposed or exposed to increasing concentrations of silica particles (100, 200, or 500 µg/mL). Notably, 4-day-old worms exposed to silica NPs, including tire wear components Aerosil 90 or Aerosil 200, showed reduced movement in comparison to controls. (**C**,**D**) Automatic quantification of thrashing tracks in (**B**) with Aerosil 90, fumed silica NPs with a diameter of 20 nm; Aerosil 200, fumed silica NPs with a diameter of 14 nm; BULK silica, silica particles with a diameter of 500–1000 nm; H_2_O, distilled water. (**C**) in 2-day-old, (**D**) in 4-day-old *C. elegans*. Statistical significance is indicated as follows: *, *p* < 0.05; **, *p* < 0.01; ****, *p* < 0.0001.

To validate the effects of silica NPs on *C. elegans* locomotion, the behavioral arena was further developed to visualize individual thrashing tracks through single-worm tracking (SWT) and automated quantification of swimming movements. Wild-type *C. elegans* (N2) were either mock-exposed (H_2_O) or exposed to precipitated silica NPs with a diameter of 50 nm, fumed silica NPs with a diameter of 20 nm (Aerosil 90) or 12 nm (Aerosil 200), or BULK silica in 96-well microtiter plates at specified concentrations and durations (Figure 3A). After exposure, worms were transferred to 6-well agar plates, and individual thrashing tracks were recorded by video microscopy (Figure 3B). Color-coded tracks visualized movement over long distances in unexposed controls (H_2_O), and GABA reporter worms exposed to 200 μg/mL BULK silica particles. However, tracks became shorter and less linear with increasing concentrations of silica NPs and age. Specifically, 4-day-old worms showed a clear decrease in movement after NP exposure, with some worms exhibiting no movement at all in a sub-cohort (Figure 3B). The comparative screening of nanomaterials with different properties indicated that at high concentrations, the tire wear components Aerosil 90 and Aerosil 200 transformed the worms into non-movers. The display of the individual thrashing tracks optimally visualizes changes in the neural behavior of worms exposed to different environmental conditions. It particularly highlights the individuality within a cohort, as even under harsh conditions, some resilient worms move longer distances, while others cease moving altogether.

Next, the recorded movements from the SWT were quantified automatically. The data showed that 2-day-old mock-exposed controls (H_2_O) exhibited a high average thrashing frequency of 134 thrashes per 30 s (Figure 3C). In contrast, worms exposed to silica NPs at concentrations of 100 µg/mL and 200 µg/mL, as well as those exposed to Aerosil 90 at 500 µg/mL, demonstrated a slight reduction in thrashing activity. Notably, worms exposed to Aerosil 200 at 500 µg/mL showed a significant decrease in thrashing (*p* < 0.05), averaging 110 thrashes per 30 s. The group treated with BULK silica exhibited a thrashing frequency similar to that of the mock-exposed control group. The results for 4-day-old worms revealed more pronounced differences in thrashing behavior (Figure 3D). The control group maintained a high average thrashing rate of 125 thrashes per 30 s. However, groups exposed to silica NPs at 100 µg/mL and 200 µg/mL showed decreased average thrashing rates of 92 and 67 thrashes per 30 s, respectively. Worms exposed to 500 µg/mL of Aerosil 90 or Aerosil 200 showed mean thrashing rates of 41 and 60 thrashes, respectively. These reductions in thrashing behavior were statistically significant (*p* < 0.001). Automatic quantification of SWT data corroborates the findings from previous manual counting, thereby confirming the concentration- and age-dependent reduction in *C. elegans* movement phenotypes by silica NPs. Additionally, automated analysis enables the systematic investigation of additional environmental variables, enhancing our understanding of silica NP effects on the neural system.

To investigate if the observed reduction in thrashing rates was due to the effects of silica NPs on the body-wall muscle tissues of *C. elegans*, we used a strain of reporter worms expressing GFP under the myosin-3 (myo-3) promoter in body-wall muscle cells. This allowed us to assess potential damage to sarcomere filaments caused by silica NP exposure. However, GFP fluorescence revealed a consistent and well-organized striated pattern of sarcomeres in both control and silica NP-exposed worms, indicating intact muscle structure (Figure S6). No disruption, disorganization, or fragmentation of sarcomere filaments in the exposed worms compared to controls was observed. These findings suggest that silica NP exposure does not cause structural damage to muscle tissues in *C. elegans*.

### 3.4. Silica NPs Target GABA Metabolic Pathways

**Figure 4:**
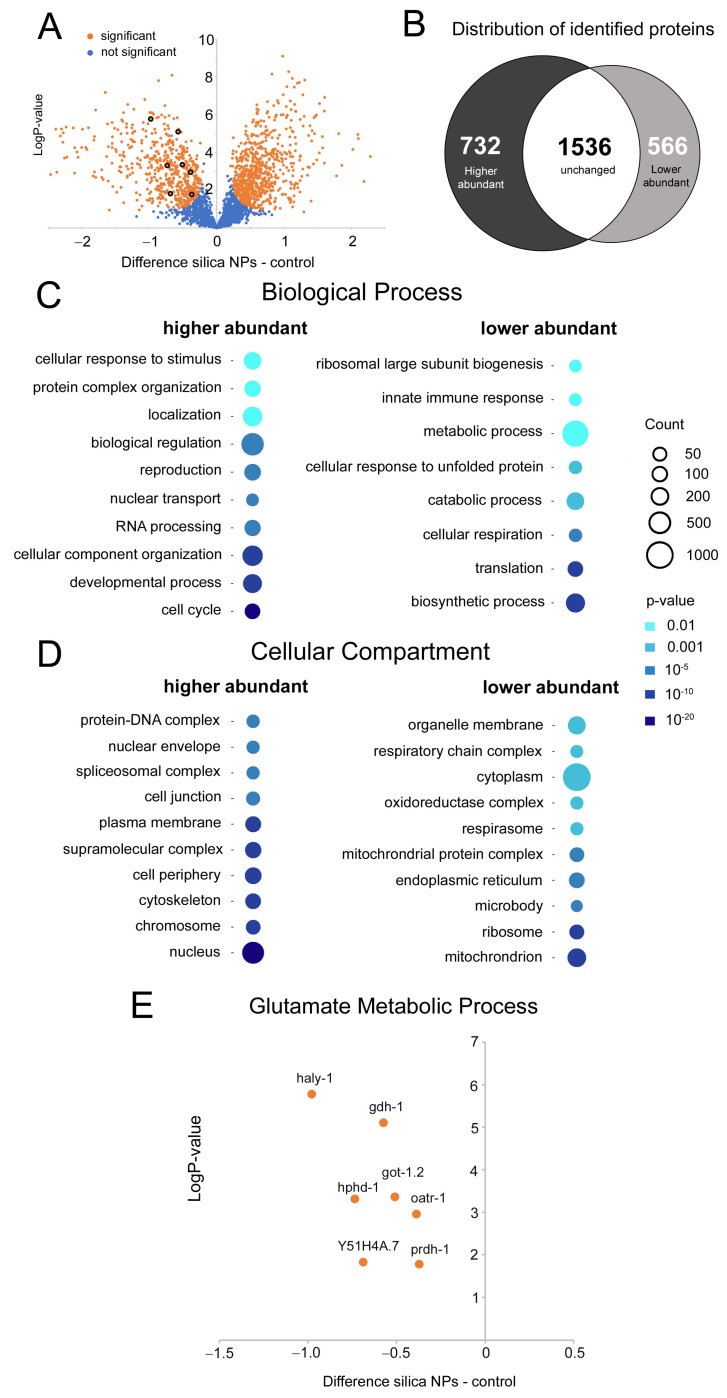
Changes in protein abundance of young *C. elegans* reporter worm for GABAergic neurons that were exposed to silica NPs. (**A**) Volcano plots representing differentially abundant proteins identified by quantitative mass spectrometric analysis. *C. elegans* reporters (strain EG1285) were mock-exposed (distilled water) or exposed to silica NPs (200 µg/mL) for 3 days. Total protein was digested with trypsin and subjected to mass spectrometry. The difference shown represents the difference in the mean values of the log_2_ transformed normalized intensities. Protein and associated gene ontologies (GOs) were categorized using databases including UniProt, PANTHER, and WormBase. Genes with a *p*-value smaller than 0.05 were considered significantly different (highlighted in orange). (**B**) Venn diagram depicting the distribution of proteins, categorized as higher abundant, lower abundant, or unchanged. (**C**) Enriched gene sets corresponding to biological processes associated with higher or lower abundant proteins. (**D**) Enriched gene sets corresponding to cellular compartments identified through GO analysis using the PANTHER database. (**E**) Graph illustrating differentially abundant proteins, specifically focusing on the glutamate metabolic process. Proteomic analysis was conducted in six independent experiments (biological replicates), with *n* = 8000–12,000 worms per condition per experiment. gdh-1, glutamate dehydrogenase 1; got-1.2, glutamate oxaloacetate transaminase 1.2; haly-1, highly abundant larval transcript 1; oatr-1, ornithine aminotransferase 1; prdh-1, proline dehydrogenase 1; Y51H4A.7, unnamed open reading frame.

The significant loss of GABAergic neurons and the associated deficits in thrashing behavior observed following exposure to silica NPs prompted an investigation into the underlying molecular pathways. A mass spectrometry-based proteomic analysis was performed to compare protein abundance profiles between the mock-exposed *C. elegans* reporters expressing GFP under the control of the *unc-47* promoter in GABAergic neurons and the respective cohort exposed to nano-silica. A volcano plot and Venn diagram summarize the distribution of identified genes in the proteomic analysis (Figure 4A,B). Out of 2834 total proteins analyzed, 566 showed a lower abundance, and 732 showed a higher abundance in response to silica NP exposure. The substantial number of proteins showing altered abundance suggests a significant impact of silica NPs, as reflected by the degeneration of GABAergic neurons, or dendrites in serotonergic and dopaminergic neurons (Figures S1–S4). Thus, silica NPs induce pan-neuronal neurodegeneration. However, the exact effects on, or interactions within, respective neuronal circuits remain to be explored.

To analyze if the changes in protein abundance can be related to specific cellular pathways, we used the PANTHER classification system for genome and gene function analysis [23] on 4-day-old worms treated with silica NPs, compared to mock controls. PANTHER analysis revealed that the proteins involved in biological processes related to ribosome biogenesis, metabolic processes, and cellular respiration show a lower abundance in silica NP-exposed worms, suggesting potential suppression of protein synthesis, energy production, and overall metabolic activity (Figure 4C,D). Notably, proteins associated with glutamate metabolism, which supports GABA synthesis, showed an overall lower abundance pattern (Figure 4E), particularly affecting glutamate dehydrogenase (gdh-1) and glutamate oxaloacetate transaminase (got-1.2). Gdh-1 is essential for converting glutamate to α-ketoglutarate, a critical step also linked to GABA synthesis. Downregulation of gdh-1 could thus limit the availability of the neurotransmitter GABA, which in turn impairs the inhibitory signaling required for coordinated locomotion and promotes neuronal hyperactivity. In mice, GDH deficiency disrupts glutamate homeostasis and excitatory/inhibitory balance, leading to impaired hippocampal-prefrontal cortex connectivity and behavioral deficits [24]. Similarly, got-1.2 is essential for converting glutamate to α-ketoglutarate and aspartate, necessary for amino acid metabolism and neurotransmitter cycling. Its downregulation disrupts this balance, further impairing glutamate processing and GABA synthesis. The reduced abundance of gdh-1 and got-1.2 may exacerbate GABA deficiency, compromising inhibitory control by GABAergic neurons and increasing neuronal stress and vulnerability to damage. Thus, the downregulation of gdh-1 and got-1.2 correlates with impaired GABA synthesis and may contribute to the degeneration of GABAergic D-type motor neurons in *C. elegans* exposed to silica NPs. Consistent with this idea, mutations that reduce GABA synthesis, such as mutations in the *unc-25* gene, which encodes for GAD, or impair GABAergic neurotransmission, lead to the degeneration and loss of GABAergic neurons [25,26]. Clinical examples in neurodegenerative diseases likewise underscore the critical role of neurotransmitter levels in maintaining neuronal health and function [27].

### 3.5. Silica NPs Reduce Global Protein Levels

The results of the GO analysis showed a significantly lower abundance of proteins involved in biosynthetic processes and translation, suggesting potential suppression of protein synthesis, energy production, and overall metabolic activity following exposure to silica NPs. To further investigate this, we compared global protein expression in 4-day-old mock-exposed versus silica NP-exposed *C. elegans* reporters of the GABAergic nervous system, using SDS-PAGE and silver staining (Figure S7). We observed a clear reduction in total protein amount in the silica NP-exposed samples, which corroborated the reduced expression of genes associated with the GO group’s translation and biosynthetic processes. Likewise, the results corroborate previous results showing that silica NPs provoke a petite phenotype that presents with small and frail body morphology [28]. Specifically, this study demonstrated that silica NPs impair OPT-2/PEP-2-dependent trafficking of nutrient peptides in the intestinal epithelium of *C. elegans*, which may in turn disrupt the supply of (di)peptides essential for translation and compromise protein synthesis.

## 4. Conclusions

Silica NPs have been shown to induce pan-neuronal degeneration in the nematode *C. elegans*, affecting the GABAergic, serotonergic, and dopaminergic nervous systems early in adult life. There is a complete loss of anteriormost and posteriormost GABAergic D-type motor neurons, highlighting the widespread and damaging effects of silica NPs on these crucial neural pathways in the model organism. Silica NP-induced neurodegeneration is accompanied by neuromuscular defects such as a reduction in crawling amplitude on solid ground and reduced swimming movement that is similar to behavior seen after ablation of GABA neurons or mutants with impaired GABA metabolism [9]. Consistently, the proteomics of 4-day-old *C. elegans* exposed to silica NPs revealed a lower abundance of proteins gdh-1 and got-1.2 that are critically involved in GABA synthesis. Taken together, this newly identified pattern of anterior- and posteriormost GABAergic D-type neuron loss, coupled with the semi-automated behavioral arena, provides a valuable system for investigating the exposome, including mixtures of pollutants such as air pollutants, and non-chemical factors.

Consistent with the One Health framework, which integrates human, animal, and environmental health, pan-neuronal degeneration of silica and other nanoparticles should be validated in vertebrate animal models and human cohorts exposed to air pollution [29,30,31]. Whole-genome association studies (GWAS) of respective cohorts may be mined for the candidate genes identified in *C. elegans* proteomes exposed to a similar exposome. This approach exploits the significant conservation of molecular pathways between humans and the model organism *C. elegans* as elegantly described by Kaletta and Hengartner [32], and serves as a critical component in the development of safe nanotechnology.

## Data Availability

The mass spectrometry proteomics data have been deposited to the ProteomeXchange Consortium via the PRIDE (PMID 34723319) partner repository with the dataset identifier PXD056717.

## Supplementary Materials

The following supporting information can be downloaded at: https://www.mdpi.com/article/10.3390/cells14030190/s1. Supplemental materials contain seven figures: Neurodegeneration in serotonergic neurons of *C. elegans* following exposure to silica nanoparticles (Figure S1), Silica nanoparticles accelerated neuromuscular defects in *C. elegans* reporters of serotonergic neurons (Figure S2), Neurodegeneration in dopaminergic neurons of *C. elegans* following exposure to silica nanoparticles (Figure S3), Silica nanoparticles accelerated neuromuscular defects in dopaminergic reporter *C. elegans* (Figure S4), Changes in protein abundance of young *C. elegans* reporter worms for GABAergic neurons after exposition to silica nanoparticles (Figure S5), Sarcomere structure remains intact in C. elegans muscles following exposure to silica nanoparticles (Figure S6), and Silica nanoparticles induce reduction in total protein amount (Figure S7).
