# Supplementary material for: Loss of γ-aminobutyric acid D-Type Motor Neurons in Young Adult Caenorhabditis elegans Following Exposition with Silica Nanoparticles
Source: Cells. 2025 Jan 27;14(3):190. doi: 10.3390/cells14030190 (PMC11816968; doi:10.3390/cells14030190)
Supplement: Supplementary file 1 [file cells-14-00190-s001.zip › cells-3394524-supplementary.pdf]

Supplementary materials

TITLE: Loss of γ-aminobutyric acid D-Type Motor Neurons in Young Adult Caenorhabditis elegans Following Exposition with Silica Nanoparticles

AUTHORS:

Dang Tri Le, Stella Pauls, Gereon Poschmann, Kai Stühler and Anna von Mikecz

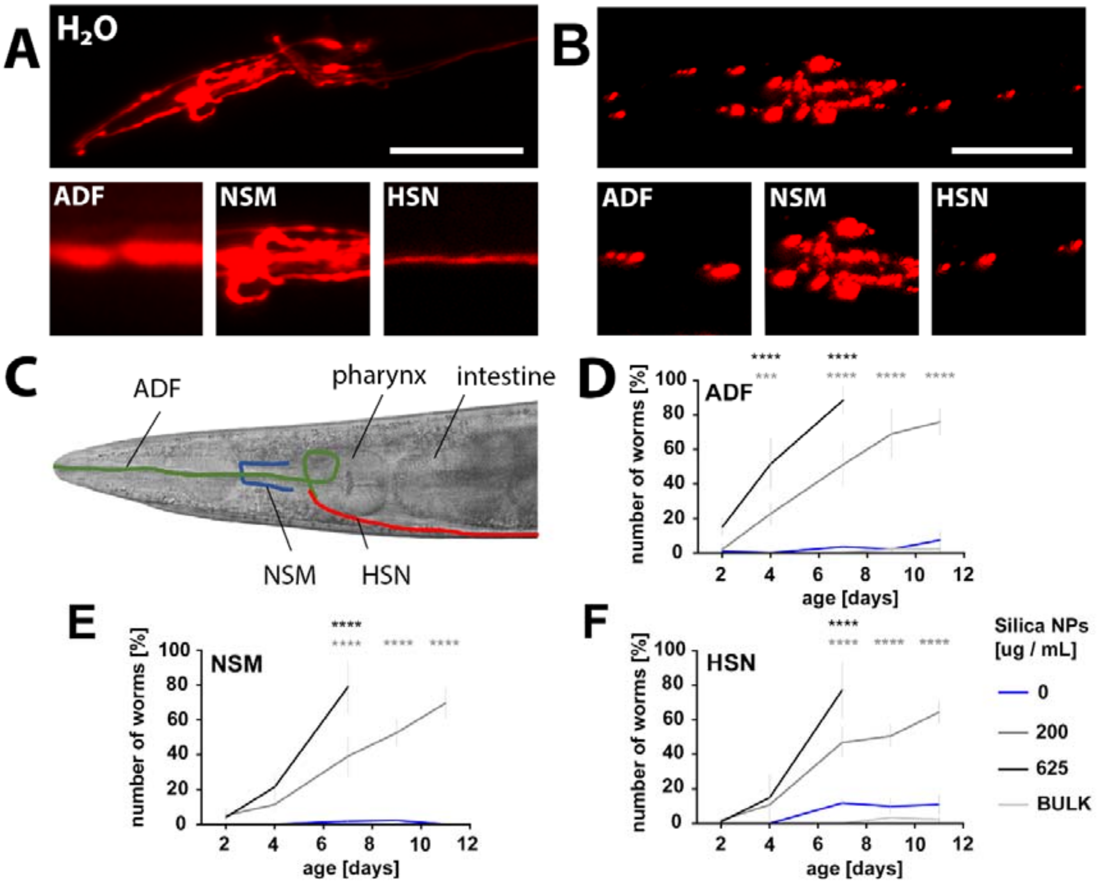

Supplementary Figure 1. Neurodegeneration in serotonergic neurons of *C. elegans* following exposure to silica nanoparticles. Representative fluorescent micrographs of 9-day-old adult *C. elegans* expressing DsRed2 under control of the tryptophan hydroxylase (*tph-1*) promotor in serotonergic neurons. (A) Reporter worms were mock-exposed (H_2O), or (B) exposed to 200 µg/mL silica NPs. The insets show magnified views of

ADF, NSM and HSN neurons. A discontinuous dotted fluorescence pattern was categorized as neurodegeneration. **(C)** Schematic representation of the *C. elegans* pharynx region with color coded locations of serotonergic neurons ADF (green), NSM (blue), and HSN (red). **(D-F)** Quantification of neurodegeneration in 2- to 11-day-old nematodes that were either mock exposed or exposed to silica NPs, and BULK silica, at the indicated concentrations. The y-axis shows percentage of worms with neurodegeneration. Values represent means ± SD from three independent experiments with n = 27–70 nematodes per condition per experiment (one-way ANOVA with Tukey's post hoc test). Scale bars, 50 μm. *, $p < 0.05$; **, $p < 0.01$; ***, $p < 0.001$; ****, $p < 0.0001$; ADF, amphid defective neuron; HSN, hermaphrodite specific neuron; NSM, neurosecretory motor neuron; SD, standard deviation.

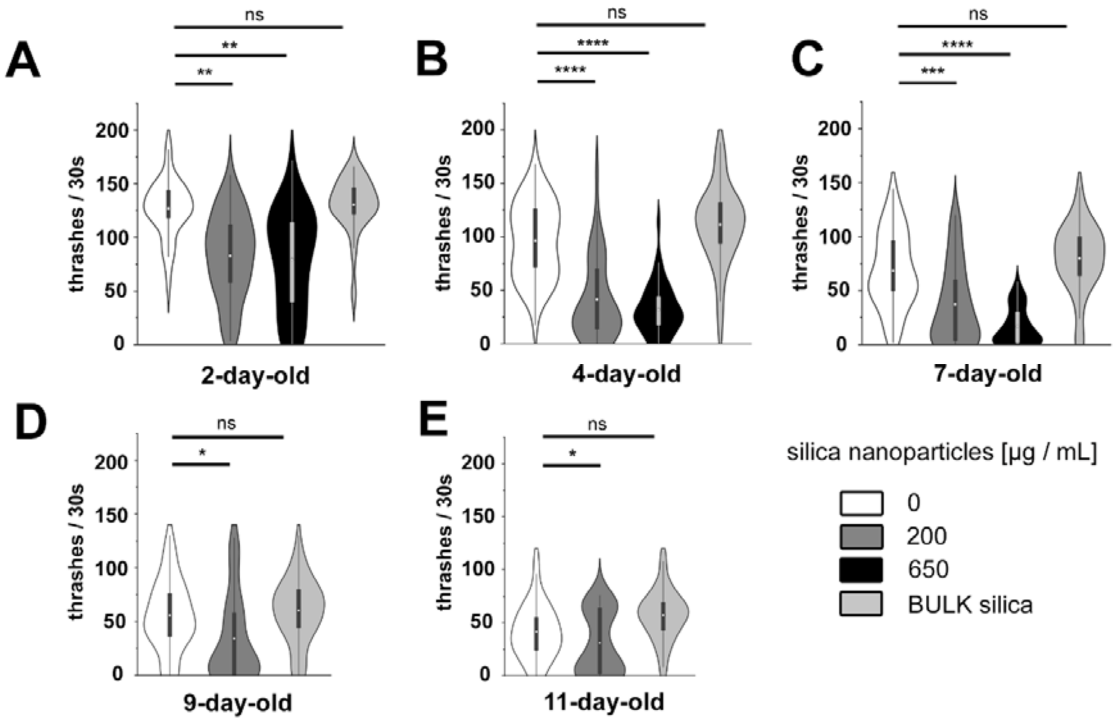

Supplementary Figure 2. Silica nanoparticles accelerated neuromuscular defects in *C. elegans* reporters of serotonergic neurons. (A–E) Reporter *C. elegans* that express DsRed2 under control of the tryptophan hydroxylase (*tph-1*) promotor in serotonergic neurons, were cultured in liquid media and fed with *E. coli* OP50. The locomotion phenotype, 'swimming', was quantified in 2- to 11-day-old nematodes that were either mock-exposed (distilled water), or exposed to the indicated conditions. (A–E) Violin plots show means ± SD

from three independent experiments with n = 15 worms per condition per experiment. *, p < 0.05; **p < 0.01; ***p < 0.001; ns, not significant; s, seconds; SD, standard deviation.

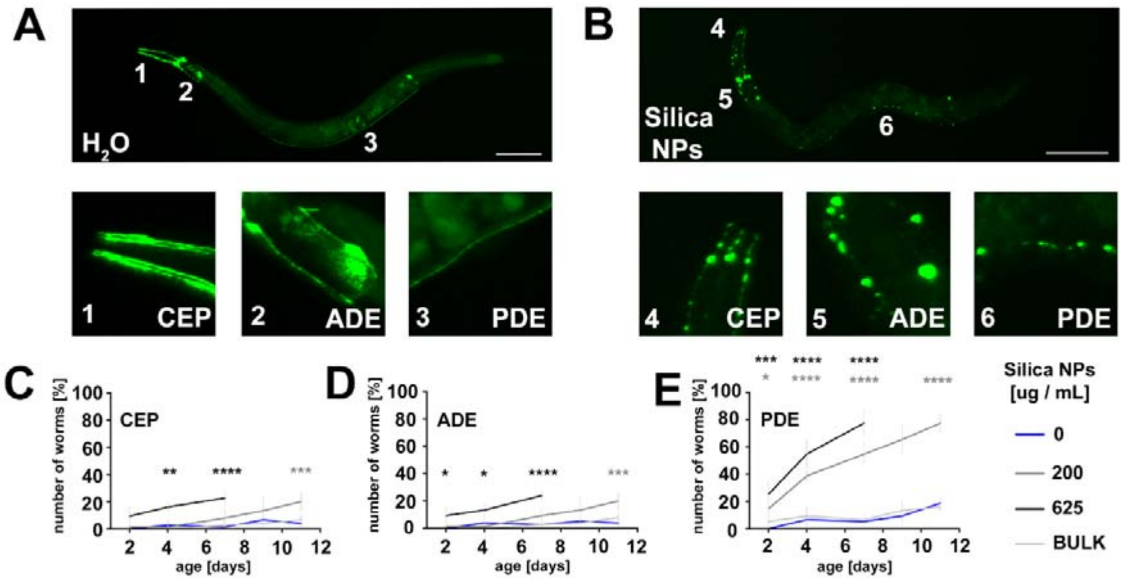

Supplementary Figure 3. Neurodegeneration in dopaminergic neurons of *C. elegans* following exposure to silica nanoparticles. Representative fluorescence microscopy micrographs of 9-day-old adult *C. elegans* stably expressing green fluorescent protein (GFP) under the control of the dopamine transporter (*dat-1*) promoter in dopaminergic neurons. (A) Reporter worms were mock-treated with H_2O, or (B) exposed to 200 µg/mL silica NPs. The insets show magnified views of neurons CEP, ADE, and PDE. A discontinuous dotted fluorescence pattern was categorized as neurodegeneration. (C-E) Quantification of neurodegeneration in 2- to 11-day-old worms that were either mock-exposed or exposed to silica particles as indicated. Values represent means ± SD from three independent experiments with n = 25-30 nematodes per condition per experiment (one-way ANOVA with Tukey's post hoc test). Scale bars, 50 µm. *, p < 0.05; **, p < 0.01; ***, p < 0.001; ****, p < 0.0001; ADE, anterior deirid neuron; CEP, cephalic neuron; ns, not significant; PDE, posterior deirid neuron; SD, standard deviation.

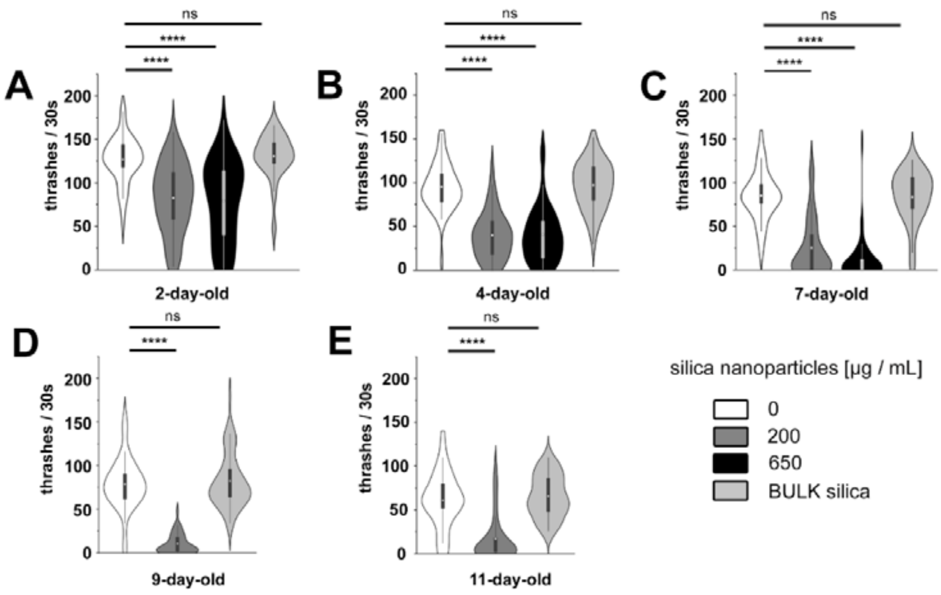

Supplementary Figure 4. Silica nanoparticles accelerated neuromuscular defects in dopaminergic reporter *C. elegans*. (A–E) Reporter *C. elegans* that express green fluorescent protein (GFP) under control of the dopamine transporter (*dat-1*) promoter in dopaminergic neurons were cultured in liquid media and fed with *E. coli* OP50. The locomotion phenotype, 'swimming', was quantified in 2- to 11-day-old nematodes that were either mock-exposed (distilled water), or exposed to the indicated conditions. (A–E) Violin plots show means ± SD from three independent experiments with n = 15 worms per condition per experiment. *, $p < 0.05$; **$p < 0.01$; ***$p < 0.001$; ns, not significant; s, seconds; SD, standard deviation.

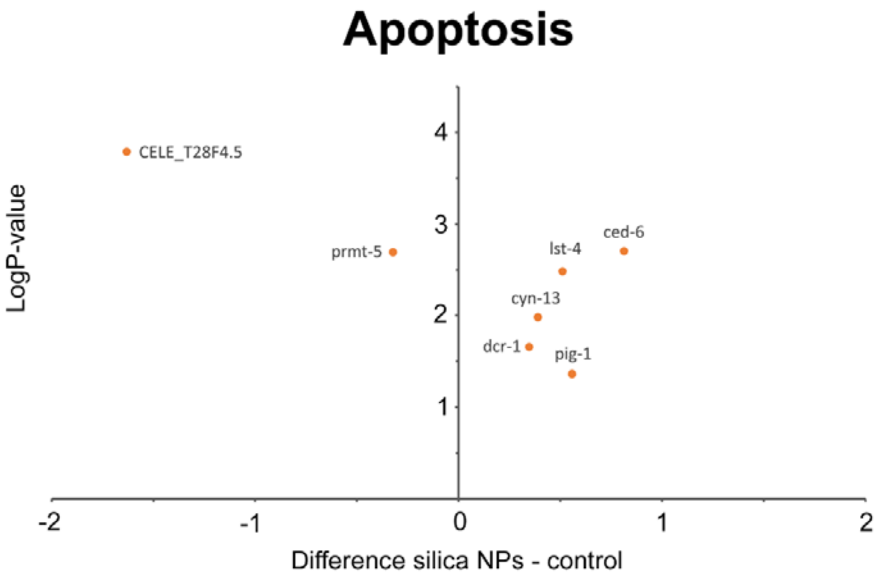

Supplementary Figure 5. **Changes in protein abundance of young *C. elegans* reporter worms for GABAergic neurons after exposition to silica nanoparticles.** Graph illustrating differentially abundant proteins, specifically focusing on the process of cell death by apoptosis. Two proteins were less abundant, and 5 proteins showed higher abundance after exposure of 4-day-old reporter worms to silica nanoparticles. Proteomic analysis was conducted in six independent experiments (biological replicates), with n = 8,000–12,000 worms per condition per experiment. ced-6, cell death abnormal 6; CELE_T28F4.5, predicted gene located on cosmid T28F4; cyn-13, cyclin-dependent kinase inhibitor 13; dcr-1, dicer-related-1; lst-4, long spliceosomal transition 4; pig-1, paralyzed isolated germline 1; pmrt-5, protein methyltransferase related 5.

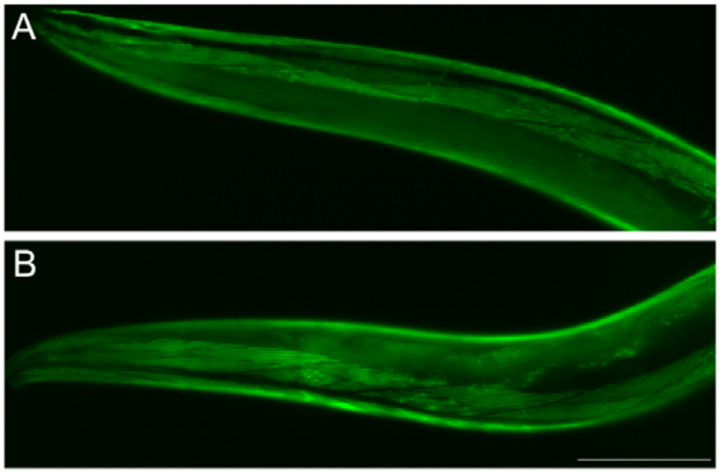

Supplementary Figure 6. **Sarcomere structure remains intact in *C. elegans* muscles following exposure to silica nanoparticles.** Representative fluorescence microscopy micrographs of 4-day-old adult *C. elegans* stably expressing green fluorescent protein (GFP) under the control of the myosin-3 promoter (*myo-3*) in body wall muscles. Reporter worms were cultured at 20 °C and fed with *E. coli* OP50, exposed to (A) mock-treatment with H_2O, or (B) silica NPs (200 µg/mL). Scale bars, 50 µm.

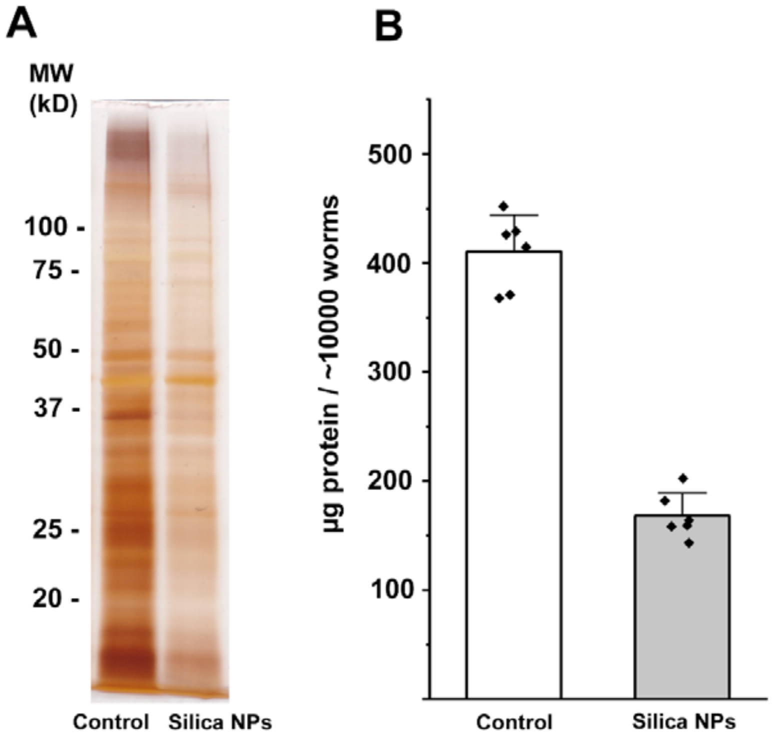

Supplemental Figure 7. Silica nanoparticles induce reduction in total protein amount. 4-day-old hermaphrodite *C. elegans* reporters for GABAergic neurons (strain EG1285) were either mock-exposed or exposed to silica nanoparticles (200 µg/mL). (A) The worms (n=8,000-10,000) were lysed, total proteins were separated by SDS-PAGE, and visualized by Silver staining (gel, right). (B) Quantification of total protein content after worm lysis. Controls show more than double protein biomass compared with silica nanoparticle-exposed worms (unpaired t-test p-value 3.6 E^{-08}).
